# Angular alignment, rotational profile, and joint range of motion in the lower limb of typically developing children from 7–16 years of age: a cross-sectional study

**DOI:** 10.2340/17453674.2025.43478

**Published:** 2025-05-01

**Authors:** Christian WONG, Michael Mørk PETERSEN, Trine HENRIKSEN, Ales JURCA, Soeren BOEDTKER, Andreas BALSLEV-CLAUSEN, Steen HARSTED

**Affiliations:** 1 Department of Orthopedic Surgery, Copenhagen University Hospital, Rigshospitalet, Denmark; 2 Copenhagen University Hospital, Hvidovre, Denmark; 3 Association of Danish Podiatrists, København S, Denmark; 4 Volumental AB, Stockholm, Sweden; 5 Jozef Stefan International Postgraduate School, Ljubljana, Slovenia; 6 Center for Muscle and Joint Health, Department of Sports Science and Clinical Biomechanics, University of Southern Denmark, Denmark; 7 Medical Spinal Research Unit, Spine Center of Southern Denmark, University Hospital of Southern Denmark, Denmark

## Abstract

**Background and purpose:**

We aimed to update reference intervals for anthropometric parameters for the passive joint range of motion (ROM), rotational profile, and angular alignment of the lower limb in typically developing children (TDC), to compare the association of the variables age, left–right sidedness, body mass index (BMI), and sex.

**Methods:**

We conducted a cross-sectional study in a convenience sample of TDC from the 1^st^, 5^th^, and 9^th^ grades (6–17 years) in a randomized selection of Danish primary schools. We examined the anthropometric parameters in a non-clinical setting. Descriptive statistics were used to characterize the data. To explore potential differences across the variables, we utilized Bonferroni-corrected Welch’s 2-sample t-tests, one-way analysis of means, and univariable linear regression.

**Results:**

We analyzed the associations between the variables and the anthropometric parameters in 501 TDC, aged 6 to 17 years. We found a statistically significant, but not clinically meaningful decrease in ROM for the hip, knee, and ankle as well as decreased femoral anteversion and increased tibial torsion with increasing age, but no association with sex or sidedness. However, several associations between BMI and ROM measurements were statistically significant and potentially clinically meaningful, with ROM decreasing by approximately 0.4° to 1.2° per unit increase in BMI, particularly for hip, knee, and ankle flexion movements.

**Conclusion:**

Anthropometric parameters remain clinically stable after 7 years of age and are affected only by the BMI but not sex or age. We found a statistically significant but not clinically relevant decrease in torsion and joint ROM with increasing age.

Knock knees, in-toeing, and flatfeet in children can elicit considerable apprehension among parents and caregivers. Comprehending the standard variations in passive joint range of motion (ROM), angular alignment, and rotational profiles in normally developing children (TDC) is crucial for differentiating natural changes from problems that may result in diseases and disabling conditions as children mature. Precise and normative data on these metrics can assist healthcare practitioners in offering clarity and reassurance to caregivers.

Previous research, notably by Staheli and associates in the 1980s and later investigations in the early 21st century, has examined these parameters [1]. Nevertheless, comprehensive data that integrates all 3 critical measures—joint range of motion, angular alignment, and rotational profiles—is still limited [2-10]. Moreover, demographic and anthropometric variables, including age, sex, laterality, weight, height, and body mass index (BMI), affect these parameters. These factors can influence physical activity, thereby heightening the risk of accidents and chronic joint health problems, including deterioration over time [11,12]. Therefore, we initiated a project for the evaluation and screening of children’s lower extremities in Danish primary schools.

We aimed to update and expand the reference material for anthropometric parameters for the joint ROM and rotational and angular status in the lower limbs of children. The study included children aged 6 to 17 years to compare the association of the variables age, left–right-sidedness, BMI, and sex.

## Methods

### Study design

The study design was a cross-sectional study that entailed an examination of TDC from Danish primary schools in a non-clinical setting. The schools were selected randomly, and we planned to enroll 1,000 children. The study plan is described in detail in a previously published protocol article that includes the study design, the organization of the research group, planned statistical analysis, the school randomization process, data processing, ethical considerations, approvals, and data registration procedures [13]. The study followed the standards for Strengthening the Reporting of Observational Studies in Epidemiology (STROBE) [14].

### Population

The children included in the study attended 1^st^ (mean age: 7.6) 5^th^ (mean age: 11.6), or 9^th^ (mean age: 15.5) grades in randomly selected Danish primary schools in the Capital Region and Region Sjaelland of Denmark. Inclusion criterion was informed signed parental consent, and exclusion criteria were not wishing to participate in the study or the presence of any coexisting diagnoses that may have musculoskeletal manifestations discovered during the study. The protocol article describes the procedure of invitation to the study, inclusion procedure, the need for treatment during and after the study, exclusion from the study, discontinuation of the study, safety endpoints, and consent to participate [13].

### Assessments

The children were examined wearing specially provided shorts by 3 teams of 2 podiatrists in 40-minute sessions. The formal training of the examiners and the test–retest are described in the protocol study [13]. The assessments of the hip, knee, and ankle consisted of anthropomorphic measurements of joint ROM of the hip (flexion, internal, and external rotation), knee (flexion and extension), and ankle joint on a flexed and extended knee in the supine position, the rotational profile (torsion of the tibia, foot–thigh angle in the prone position, and femoral anteversion in the supine position) and angular alignment of the knee and ankle standing. We examined both legs as in previous studies [1,5-10,15,16]. The children were examined in a standardized manner and using an electronic goniometer when feasible or otherwise a protractor. The examinations are described in our protocol article [13], and are shown with links to videos in the Supplementary data.

We performed a test and retest analysis with a subset of 50 children as recommended for test–retest studies, where we found that reliability analysis had poor to moderate results for all other measurements (ICC < 0.5, ICC < 0.75) and outside limits of agreement [17-19].

### Statistics

The distribution of all variables was examined graphically using density plots. Anthropomorphic data on age, sex, height, weight, and BMI were stratified according to school grade and presented using mean and standard deviation (SD). Reference intervals were calculated using the 2.5^th^ and 97.5^th^ percentiles. The clinical measures of joint ROM, rotation, and angular profiles were compared for differences in sidedness using Welch 2-sample t-tests. These clinical measures were then stratified according to school grade and were presented using means and SDs [10]. Differences between age and sex were examined using a one-way analysis of means or Welch’s 2-sample t-tests. The associations between BMI and the lower extremity measures were analyzed using univariable regression analyses with BMI as the independent variable. Given the multiple comparisons in this study, we applied a Bonferroni correction to control for the increased risk of Type I error. The corrected significance level was calculated by dividing the original alpha level (0.05) by the number of comparisons. However, in the analysis of sidedness, the purpose was to validate the use of data from only 1 side. This was not a multiple-hypothesis test so applying a correction such as Bonferroni was not relevant. The analysis was conducted using R (R Foundation for Statistical Computing, Vienna, Austria), and the following R packages: tidyverse, gtsummary, ggpubr, and quantreg [20].

### Ethics, data sharing plan, funding, use of AI, and disclosures

We applied to the local ethics committee for ethical approval (H-22002997), and the study was not appropriate for evaluation for approval following the Danish Act on Research Studies § 2. Regional registration was obtained following the Danish Data Protection Agency as stipulated by Danish law J.nr. 2008-41-2240, and we adhered to the relevant guidelines and were approved by the local review board (Privacy). The study adhered to relevant national guidelines and was carried out following the Declaration of Helsinki. Informed signed consents have been obtained from all subjects’ legal guardians. Data sharing will be considered upon written request. AI tools were not used in our submission. This study is funded by the Association of Danish Podiatrists with the sum of DKK610,000. All authors declare no conflicts of interest except Ales Jurca, who is employed by Volumental AB. The funders had no role in the design of this study; in the collection, analysis, or interpretation of the data; in the writing of the manuscript; or in the decision to publish the results. Complete disclosure of interest forms according to ICMJE are available on the article page, doi: 10.2340/17453674.2025.43478

## Results

**Figure 1:**
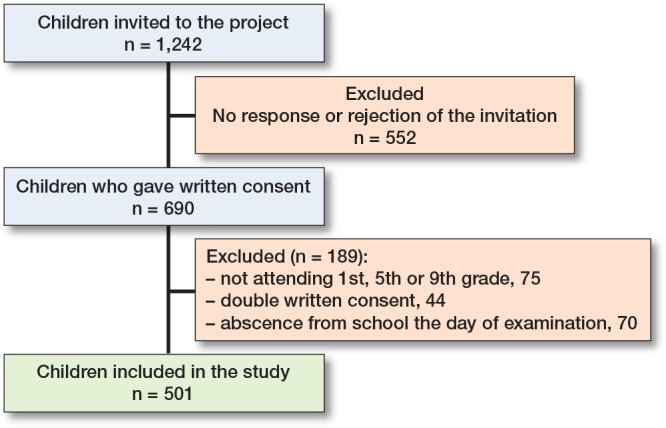
Inclusion process with the number (n) of children invited to the project (1,242). Parental consent was obtained from 690 children and 501 were examined at the start of the project.

20 schools were selected randomly according to their socioeconomic profiles and straight-line distance. Figure 1 shows the inclusion process.

**Figure 2:**
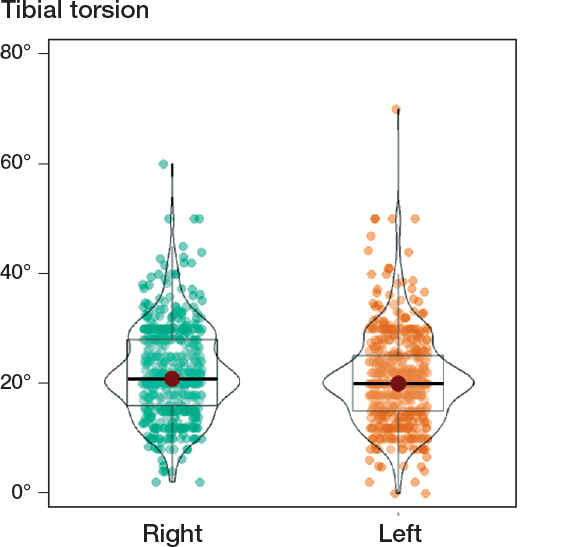
Tibial torsion for the left and right legs of the examined children.

**Figure 3:**
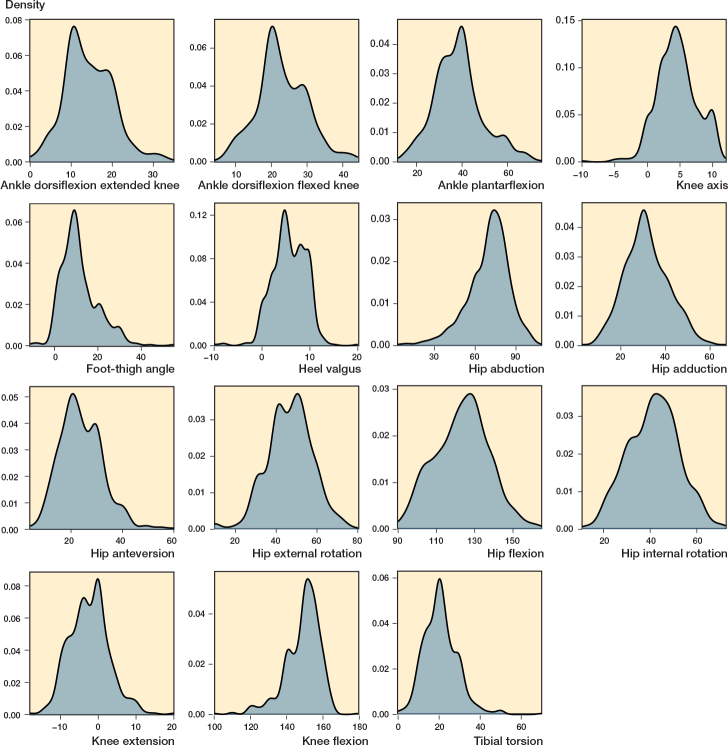
Distribution plots for the clinical assessments showing the left side.

We included 501 children: 203 in the 1^st^ grade (391 invited), 211 in the 5^th^ grade (410 invited), and 87 in the 9^th^ grade (441 invited). The sex ratio (boy:girl) was ~1:1 (242:259). The children’s age range was 6.8–16.5 years, height range 121–182 cm, and weight range 22–75 kg (SD 16) (Table 1). As seen in Figure 2 (see Appendix), we found a median tibial torsion of 20.0° and 20.9° on the right and left sides, respectively. Concerning sidedness, this was the only statistically significance difference (P = 0.009). Figure 3 and Table 2 (see Appendix) show the distribution plots and results for sidedness.

**Table 1:** Distribution of subjects’ age, sex, height, weight, and body mass index (BMI) according to school grade. Values are mean (SD) or count (%)

Item	Overall n = 501	1^st^, n = 203	School grade 5^th^, n = 211	9^th^, n = 87
Age	10.7 (2.9)	7.6 (0.43)	11.6 (0.42)	15.5 (0.39)
Sex, n (%)				
Girls	242 (48)	103 (51)	100 (47)	39 (45)
Boys	259 (52)	100 (49)	111 (53)	48 (55)
Height, cm	147 (17)	129 (5)	153 (7)	173 (8)
Weight, kg	41 (16)	27 (5)	45 (10)	64 (12)
BMI	18.3 (3.6)	16.2 (2.1)	19.1 (3.4)	21.4 (4.0)

**Table 2:** Distribution of mean angular alignment, rotational profiles, and range of motion for left-/right-sidedness with standard deviation (SD)

Item	Side		P value^a^
	dexter n = 501	sinister n = 501	
Hip flexion	124 (15)	123 (14)	0.8
Hip internal rotation	42 (12)	41 (11)	0.2
Hip external rotation	47 (12)	47 (11)	0.6
Hip abduction	69 (15)	70 (16)	0.5
Hip adduction	32 (10)	32 (10)	0.8
Knee flexion	148 (10)	149 (10)	0.5
Knee extension	–2.3 (5.1)	–2.4 (5.2)	> 0.9
Ankle dorsiflexion			
flexed knee	23 (7)	23 (7)	0.8
extended knee	14 (6)	14 (6)	0.8
Ankle plantar flexion	38 (10)	39 (11)	0.7
Hip anteversion	25 (9)	24 (8)	0.2
Axis knee	4.5 (3.1)	4.6 (3.1)	0.7
Tibial torsion	22 (8)	21 (8)	0.009
Foot–thigh angle	12 (9)	11 (8)	0.6
Heel valgus	5.3 (3.5)	5.7 (3.5)	0.12

a Welch 2-sample t-test.

### Range of motion, anatomical regions, and torsion

For the hip region, we found statistically significant differences in ROM across school grades, with lower mean values observed in older children. Specifically, hip flexion decreased by approximately 5° (q = 0.009), and internal rotation by 11° (q < 0.001) from 1^st^ to 9^th^ grade.

For the knee, flexion ROM showed a statistically significant decline across school grades, with a mean difference of 7° from 1^st^ to 9^th^ grade (q < 0.001).

For ankle dorsiflexion, we observed statistically significant differences across school grades for both measurements. Dorsiflexion with the knee flexed decreased by 3° from 1^st^ to 9^th^ grade (q < 0.001), and dorsiflexion with the knee extended decreased by 3° as well (q = 0.004).

For the rotational profile (Table 3), hip anteversion decreased significantly by 4° from 1^st^ to 9^th^ grade (q < 0.001). Post hoc comparisons showed that this decrease occurred primarily between the 1^st^ and 5^th^ grades, with no significant difference between the 5^th^ and 9^th^ grades. No significant differences were observed for the axis of the knee, tibial torsion, foot–thigh angle, or heel valgus after Bonferroni correction.

**Table 3:** Mean and standard deviations (SD) for passive ranges of motion (ROM), rotational profiles, and angular alignments according to school grade

Item	School grade			P^a^	q-value^b^
	1^st^ n = 203	5^th^ n = 211	9^th^ n = 87		
Hip flexion	126 (15)	122 (15)	121 (11)	< 0.001	0.009
Hip rotation					
internal	47 (11)	41 (12)	36 (12)	< 0.001	< 0.001
external	48 (11)	47 (12)	44 (12)	0.006	0.09
Hip abduction	69 (15)	70 (14)	67 (16)	0.3	> 0.9
Hip adduction	33 (10)	31 (10)	31 (11)	0.03	0.5
Knee flexion	151 (8)	148 (10)	144 (10)	< 0.001	< 0.001
Knee extension	–2.9 (5.5)	–1.7 (4.4)	–2.5 (5.5)	0.04	0.6
Ankle dorsiflexion					
flexed knee	24 (7)	23 (7)	21 (7)	< 0.001	< 0.001
extended knee	16 (6)	14 (6)	13 (5)	< 0.001	0.004
Ankle plantar					
flexion	39 (9)	38 (10)	39 (10)	0.5	> 0.9
Hip anteversion	27 (9)	23 (9)	23 (8)	< 0.001	< 0.001
Axis knee	4.4 (3.2)	4.7 (2.7)	4.1 (3.3)	0.2	> 0.9
Tibial torsion	21 (8)	22 (8)	24 (8)	0.02	0.2
Foot–thigh angle	11 (9)	12 (8)	11 (8)	0.8	> 0.9
Heel valgus	5.6 (3.7)	5.5 (3.3)	4.4 (3.4)	0.02	0.3

a One-way analysis of means (not assuming equal variances).

b Bonferroni correction for multiple testing.

### Other factors

In general, we found no significant differences in ROM or alignment between sexes after correction for multiple testing. However, hip internal rotation was slightly higher in girls than boys (q = 0.04), though this difference was not present when stratified by school grade.

**Figure 4:**
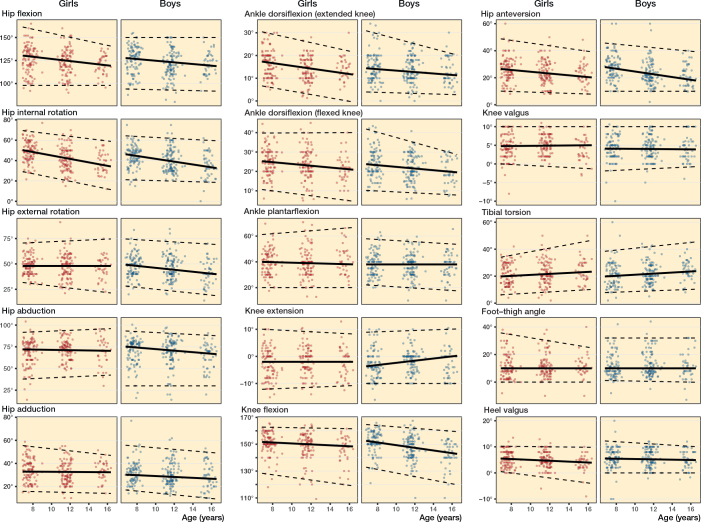
Age-related reference intervals (dashed lines: 2.5th to 97.5th percentiles) for passive range of motion for both sexes for the hip (left panels – flexion internal and external rotation, abduction and adduction); knee and ankle (middle panels – flexion and extension/plantar and dorsiflexion on straight and bent knee); and the rotational profile (right panels – femoral anteversion, knee axis, foot–thigh, ankle, and tibial torsion).

Figure 4 illustrates the ROM of the hip, knee, and ankle, as well as the rotational profile, across increasing age. The dashed lines represent lines of best fit for the 2.5^th^ and 97.5^th^ percentiles estimated using quantile regression. These lines depict the boundaries within which 95% of the data points are expected to fall. The solid line represents the line of best fit for the median, which was also estimated using quantile regression.

Tables 2 and 4 (see Appendix) show the distribution according to sex for each age group and the change in each clinical parameter (Beta) for 1 unit increases in BMI, respectively.

**Table 4:** Univariable regression analyses for passive ranges of motion (ROM), rotational profiles and angular alignment results with mean changes (Beta) for 1 unit increases in BMI

Variable	n	Beta (CI)	P	q-value^a^
Hip flexion	499	–0.94 (–1.3 to –0.59)	< 0.001	< 0.001
Hip internal rotation	499	–0.70 (–0.99 to –0.42)	< 0.001	< 0 .001
Hip external rotation	499	–0.37 (–0.65 to –0.09)	0.01	0.02
Hip abduction	497	0.07 (–0.30 to 0.43)	0.7	0.8
Hip adduction	497	–0.32 (–0.57 to –0.08)	0.01	0.02
Knee flexion	499	–1.20 (–1.4 to –0.97)	< 0.001	< 0.001
Knee extension	491	–0.02 (–0.14 to 0.11)	0.8	0.8
Ankle dorsiflexion				
flexed knee	500	–0.62 (–0.79 to –0.45)	< 0.001	< 0.001
extended knee	500	–0.41 (–0.56 to –0.27)	< 0.001	< 0.001
Ankle plantar flexion	500	0.10 (–0.15 to 0.34)	0.4	0.5
Hip anteversion	499	–0.27 (–0.49 to –0.04)	0.02	0.03
Axis knee	500	–0.04 (–0.11 to 0.04)	0.3	0.5
Tibial torsion	499	–0.09 (–0.30 to 0.11)	0.4	0.5
Foot–thigh angle	497	–0.08 (–0.29 to 0.13)	0.5	0.5
Heel valgus	499	–0.16 (–0.24 to –0.08)	< 0.001	< 0.001

CI = 95% confidence interval

a Bonferroni correction for multiple testing

## Discussion

The study reviews and expands upon the passive joint ROM, rotational profiles, and angular status in the lower limbs of individuals aged 6 to 17 years. This dataset, comprising the largest cohort for these parameters in this age group, is instrumental for clinical evaluation in pediatric musculoskeletal evaluation. We showed that clinically relevant changes do not occur from 7 to 16 years of age. Sex, age, and sidedness were not statistically significant for the examined parameters. However, several associations between BMI and ROM measurements were statistically significant and potentially clinically meaningful, with ROM decreasing by approximately 0.4° to 1.2° per unit increase in BMI, particularly for hip, knee, and ankle flexion movements. Furthermore, we observed statistically significant differences from 1^st^ to 9^th^ grade, including decreases in femoral anteversion, and flexion at the hip, knee, and ankle, as well as hip internal rotation. However, these differences were generally small relative to the overall range of each parameter and were not considered clinically meaningful. We also found lower extremity flexion with an increase in BMI; an increase of 1 unit in BMI was significantly associated with reduced ROM in hip flexion (β = –0.94), hip internal rotation (β = –0.70), external rotation (β = –0.37), adduction (β = –0.32), knee flexion (β = –1.2), ankle dorsiflexion with flexed (β = –0.62) and extended knee (β = –0.41), hip anteversion (β = –0.27), and heel valgus (β = –0.16). No significant associations were found for knee alignment or torsional measures. Previous studies also indicate that childhood and adolescent obesity induce biomechanical differences, i.e., during high-impact activities when running, jumping, and hopping [11,12].

### Hip joint

The hip joint showed a decrease in femoral anteversion with age, consistent with prior studies. Jacquemier et al. (2008) measured the rotational profile and found a decrease from 20° to 13° [5]. In our study, we found a smaller decrease from 27° to 24° for femoral anteversion, thus a greater anteversion and a lower decrease. Staheli (1985) measured internal rotation and found a decrease of 1.8° for boys and 3.8° for girls [9]. The decrease was smaller than our data (47° to 41°), and Staheli also found a marked variation between the sexes. We were unable to confirm this in our population. Changes in hip ROM, including flexion, internal rotation, and adduction, were affected by BMI, with statistically significant decreases observed for every unit increase in BMI. This aligns with previous studies linking obesity with restricted hip motion during high-impact activities, potentially increasing injury risk [11,12].

### Knee joint

For the knee joint, we observed minimal changes in angular alignment across age groups, with a tibiofemoral angle of 4.4°, which is similar to that reported by Kaspiris et al. (2013) for a similar age group [8]. Knee flexion ROM decreased with age, consistent with the literature [4,8], though the magnitude of change was not clinically meaningful. BMI-adjusted analysis showed an association between higher BMI and reduced knee flexion, potentially indicative of mechanical adaptations to increased bodyweight. The interrater reliability for knee axis measurements was poor, reflecting challenges in consistent assessment.

### Ankle joint

At the ankle, dorsiflexion ROM on both flexed and extended knees decreased with age, aligning with prior research. Lee et al. (2013) reported similar trends but with slightly higher reductions with age [3]. Our results also demonstrated that higher BMI was statistically significantly associated with decreased dorsiflexion (P > 0.001, Beta ~0.5), consistent with studies highlighting the biomechanical challenges posed by excess bodyweight during weightbearing activities [11,12]. The interrater reliability for ankle plantar flexion was poor, yet the broader estimated reference intervals mitigated the potential for overdiagnosis in normal children.

For all ranges of motion, Mudge et al. (2014) [10] found similar ROM to our study for external hip rotation, knee extension, and ankle dorsiflexion on extended and flexed knees, but lower values for hip internal rotation and hip abduction in 17 volunteers (aged 8–11) and 16 volunteers (aged 12–16), whereas Lee et al. (2013) [3] found a greater annual change in ankle dorsiflexion (0.35° in our data vs. ~ 0.7° in Mudge et al.) [10]. Sankar et al. (2012) found similar values and changes concerning age for hip ROM for hip flexion, extension, abduction, and adduction [2].

### Angular profile

For alignment, Heath and Staheli (1993) examined the angular alignment of the knee and found a decrease of 1.8° [6]. In our study, we found almost no difference (0.3°). Kaspiris et al. (2013) found a tibiofemoral angle of 4.2°, whereas we found 4.4° in 203 children of similar age [8]. The findings of our study, despite its limitations, are relevant to Denmark and may be regarded as indicative of broader patterns observed in Europe and North America. Nonetheless, their particular health markers and behaviors may not be immediately applicable to other contexts owing to variations in health systems, social structures, and cultural norms such as weight and concerns around body image [21]. Danish youngsters demonstrate comparatively elevated rates of weight-reduction practices relative to other nations, suggesting that the BMI may be lower in our community. Moreover, children from ethnic minority backgrounds in Denmark exhibit elevated rates of obesity, type 1 diabetes, hyperglycemia, and vitamin D deficiency as they engage less frequently in sports and physical activities than their ethnic Danish counterparts [22].

### Limitations

This study’s limitations include potential selection bias, as half of the eligible participants declined to participate, particularly the older age group with a dropout of four-fifths. Additionally, our reliance on manual measurements introduces variability, as indicated by our internal validity (test–retest reliability), though this reflects real-world clinical practices. In this study, we utilized age segments for the longitudinal changes that are pragmatic due to the practical necessity of the study, i.e., COVID-19, and financial constraints. Thus, we were unable to include the planned number of subjects. This also implies that we generalize from 3 different time points to reflect longitudinal changes, which is a limitation due to the current study design. We have shown data variation with mean and standard deviations, as these have been suggested as normal ranges in the statistical and clinical literature, respectively [1,10,23,24]. These are currently used as decision limits but are statistically normative values utilized to motivate surgical interventions, where there is a misconception of “normal ranges” in medical tests as absolute indicators of health or disease. Normality is dynamic, varies with individual factors, and requires contextual interpretation, to which we hope this study contributes [23,24]. In the analysis and interpretation of data in this study and its conclusions, the generalizability relies on the specificity of the evaluation methods and sample size. Our calculated reliability coefficients using intra-class correlations were in general slight and fair, but poor for knee axes and moderate for dorsal flexion of the ankle with the extended knee (0.54 and 0.58), right-sided hallux valgus (0.52), foot–thigh angle (0.66 and 0.74), right-sided hip adduction (0.58), hip anteversion (0.53 and 0.54), right-sided hip flexion (0.50), right-sided hip internal rotation (0.56), and flexion of the knee (0.58 and 0.60) and otherwise poor (ICC < 0.50), thus we believe that our results if utilized in decision-making in treatment should not be based solely on range of motion testing and clinical evaluation but assisted by radiography, gait analysis, and other tests [18]. The total planned number of included children was 1,000 children. The sample size was chosen based on a balance between the desired statistical robustness and the practical constraints of the study [23,24]. Normative data from this and other studies would suggest changes occur for important parameters before the age of 7, and our data also confirms that these anthropometric parameters remain clinically stable, meaning that “what you observe is what you can expect after 7 years of age” [1,6]. In our study, we found it meaningful to examine schoolchildren (aged 6–17) as interventions typically and at the earliest are carried out then. There are impediments as described, which make it more difficult to generalize these normative data. Nevertheless, there were no statistically significant differences identified between the subjects and dropouts in terms of their distribution across class grades. When comparing our anthropometric parameters with other normative data, this is also influenced by the evaluation method as demonstrated by Mudge et al. (2013); as an example, the rotational profile was performed in the prone position by Staheli, whereas we examined this supine as described by Netter, thus measuring bias influence both internal and external robustness [1,10]. The use of many examiners results in variability in the reliability of our assessments, as expressed in test–retest reliability, internal consistency, and interrater reliability, and indicated by the intra-class correlation coefficients (ICCs). However, the presence of variations among examiners leads to broader estimated reference intervals, reducing the likelihood of normal youngsters falling outside these intervals, and our data concurs with previous studies, thus inter-examiner variation has not influenced our data.

Malignancies, infections, or acute medical conditions warranting treatment were addressed promptly and excluded. However, we did not address previous fractures, and we did not exclude subjects with a history of previous lower extremity trauma or fractures. These injuries could potentially impact the range of motion (ROM) and confound the analysis.

### Conclusion

Anthropometric parameters remain clinically stable after 7 years of age. We found a statistically significant but not clinically relevant decrease in torsion and joint ROM with increasing age. In our larger pediatric population, our data for joint ROM, angular alignment, and rotational profile in the lower extremities are aligned with previous studies.

## Notes

The authors would like to thank nurse Christina Ystroem Bjerge and are grateful for her hard work in coordinating the collection of data.

AB-C: conceptualization, methodology, software, formal analysis, investigation. AJ: conceptualization, methodology, formal analysis, resources, data curation, writing—review and editing, visualization. CW: conceptualization, methodology, validation, formal analysis, investigation, resources, data curation, writing—original draft, writing—review and editing, visualization, supervision, project administration, funding acquisition. CYB: conceptualization, methodology, software, validation, formal analysis, investigation, resources, data curation, writing—original draft, writing—review and editing, visualization, supervision, project administration, funding acquisition. MMP: formal analysis, data curation, writing—original draft, writing—review and editing, visualization. SB: conceptualization, methodology, formal analysis, investigation, writing—review and editing. SH: software, validation, formal analysis, data curation, writing—review and editing.

Handling co-editor: Ilkka Helenius

*Acta* thanks Hanne Hedin, Antti Joonas Stenroos, and another anonymous reviewer for help with peer review of this manuscript.
